# A novel three-dimensional model for evaluation of unicompartmental knee prostheses tibial component positioning

**DOI:** 10.1007/s00402-025-05903-y

**Published:** 2025-05-03

**Authors:** Julia Elisabeth Lenz, Moritz Kaiser, Volker Alt, Michael Worlicek, Philipp von Roth

**Affiliations:** 1 University Medical Center Regensburg, Regensburg, Germany; 2 Dr. kaiser & kollegInnen MVZ GmbH, Regensburg, Germany; 3 Schulthess Klinik, Zurich, Switzerland; 4 Sporthopaedicum Straubing, Straubing, Germany

**Keywords:** 3D model, Unicompartmental Knee Prosthesis, Tibial Component, Positioning, Arthroplasty

## Abstract

**Background and objective:**

This study aimed to develop an innovative method to assess the optimal positioning of the tibial component in unicompartmental knee arthroplasty. Furthermore, the authors performed a pilot study on tibial bone resections after having performed the tibial saw cuts of unicompartmental knee prostheses in 0° and 3° in the coronal plane.

**Materials and methods:**

The study’s objective was achieved by devising a surface matching technique that involved comparing the tibial bone resections with three-dimensional Computer-Aided Design models of the selected tibial component. 44 bone resections of patients who had undergone unicompartmental knee arthroplasty with tibial resection angles of 0° and 3° varus in the coronal plane were evaluated in the pilot study. Furthermore, the possibility of evaluating the biological quality of the resection surface by examining the percentage of sclerotic bone in the most distal cut surface of the bony specimen was analyzed.

**Results:**

A new method to evaluate bony resections using a three-dimensional Computer-Aided Design model could be implemented. The implementation study including 44 tibial resections following unicompartmental knee arthroplasty did not reveal significant differences between the cohorts.

**Conclusion:**

In conclusion, we believe that the 3D modeling technique presented holds significant potential and merits application to a larger cohort of tibial bone resections for a more comprehensive investigation into the optimal tibial resection plane. Moreover, this methodology could be instrumental in the development of patient-specific implants.

## Introduction

If an individual suffers from isolated medial gonarthrosis, one potential treatment option is the implantation of a unicompartmental knee arthroplasty (UKA). Patients who undergo UKA tend to report higher satisfaction rates compared to those who undergo total knee arthroplasty (TKA) [1]. On the other hand, TKA has demonstrated better long-term durability than UKA [2]. Among the common reasons for failure in UKA, loosening of the tibial component ranks high [3]. While numerous scientific studies have focused on the optimal positioning of TKA components, the ideal positioning of UKA components requires further discussion [4], [5].

According to the manufacturers’ guidelines, the tibial component of UKA should be implanted at a 90° angle to the longitudinal axis of the tibia. However, the natural knee joint exhibits a varus tilt of approximately 3° on the medial tibial plateau [6]. In TKA, it has been found that positioning the tibial component with a slope of around 3° towards the medial side yields better outcomes [7]. The inquiry regarding whether the tibial component of the partial prosthesis should be implanted with a slight slope towards the medial side has received limited investigation thus far. Vasso et al. are among the few researchers who have explored the implantation of the tibial component at 3° varus. They demonstrated a higher survival rate for these implants compared to those implanted at 0° [7]. In a noteworthy study, Sekiguchi et al. conducted a computer simulation in 2019 to determine the optimal alignment [8]. Their findings indicated that a slight varus implantation in the coronal plane was preferable. To the best of our knowledge, no in vivo data exist on this particular topic.

Our study aimed to develop a novel method for assessing the optimal positioning of the tibial component in UKA. Furthermore, we performed a pilot study by analyzing the bone resections after performing the tibial saw cuts at 0° and 3° varus angles in the coronal plane. We hypothesized that a 90° resection in the frontal plane would result in a wedge-shaped resection due to the preoperative medial slope of the joint line. In contrast, a 3° varus resection would lead to a square-shaped resection, aligning better with the square frontal plane of the tibial component in UKA and allowing for a more natural distribution of forces. Conversely, a wedge-shaped resection would cause medial overstuffing. Furthermore, the possibility of evaluating the biological quality of the resection surface by examining the percentage of sclerotic bone in the most distal cut surface of the bony specimen was analyzed.

## Materials and methods

The primary objective was to conduct a “best-match analysis” between the bony specimens, which are produced by tibial resection for the UKA, and the CAD models of the tibial component. This analysis was carried out using the “Amira” software, version 2021.2. After creating a three-dimensional (3D) model of the tibial bone resection, we assessed the degree to which the surface of the bone resection matched the surface of the tibial component.

**Fig. 1:**
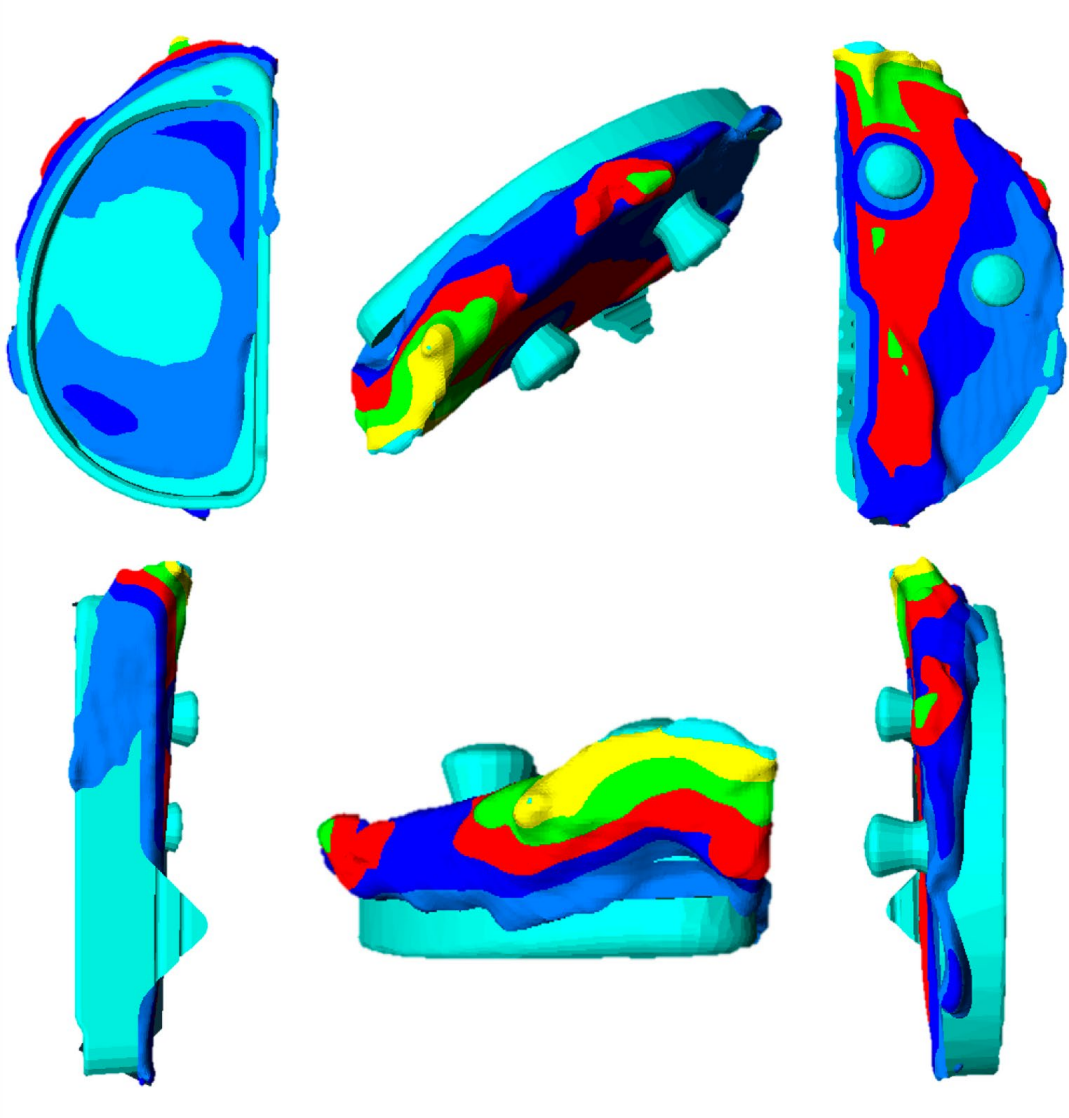
Overlay of Bone Resection and CAD-Model in six perspectives. Color Code of distance between voxels: Light Blue = 0–1, Dark Blue = 1–2, Red = 2–3, Green = 3–4, Yellow = 4–5, Turquoise = 5–6, Orange = 6–7, Pink = 7–8

In Amira, the CT and CAD model data sets were uploaded, and the bony specimens or CAD models were marked using the “Interactive Thresholding” command. Next, the bone resection or CAD model was cropped using the “Crop Editor” to eliminate any interfering factors such as the preservation box. Surface models were generated from both bodies using the “Generate Surface” command and aligned as accurately as possible using the “Align Surfaces” command. We then calculated the distances between the surfaces as well as the body volume. The 3D view provided a visualization of the distance between the resection and the CAD model using the “Surface View” command (refer to Fig. 1).

**Fig. 2:**
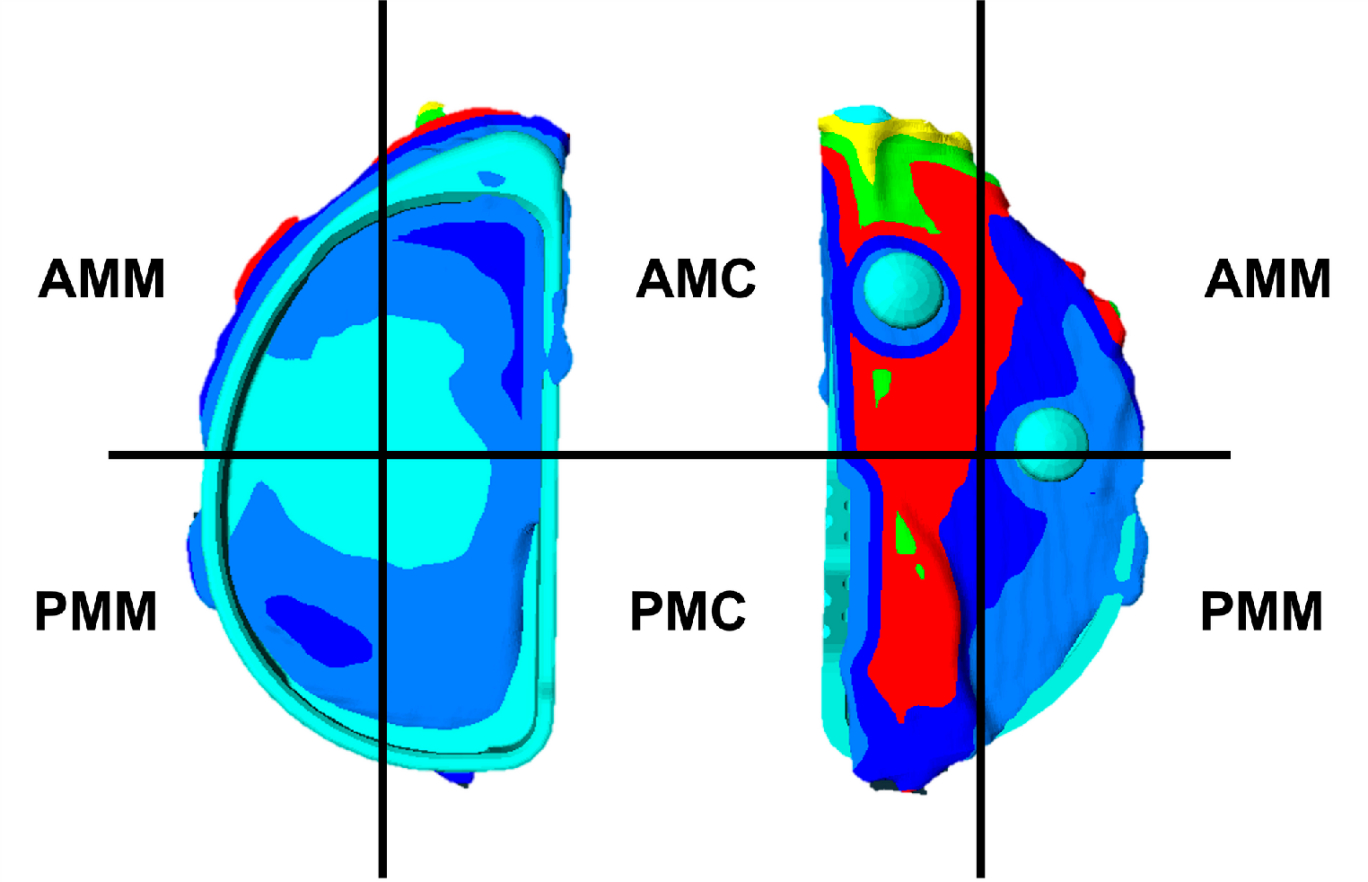
Division into compartments, based on the classification by Krause et al. (13) AMM = Anteromedial-Medial, AMC = Anteromedial-Central, PMM = Posteromedial-Medial, PMC = Posteromedial-Central

Standardized screenshots were captured from strictly anterior, posterior, and lateral views, along with at least two oblique images. These images were subsequently assessed by a single blinded rater using the tibial plateau fracture classification system developed by Krause et al. [9]. The model was divided into four compartments: Anteromedial-Medial (AMM), Anteromedial-Central (AMC), Posteromedial-Medial (PMM), and Posteromedial-Central (PMC) (refer to Fig. 2). The highest elevation of the bone above the CAD model was measured based on the anterior and posterior images. The measurements for the respective compartments were then combined, for example, AMM anterior + AMM posterior = AMM total. Lastly, the ratio between the surplus in the medial and central compartments was determined and evaluated.

For the initial investigation of our proposed method, we conducted a prospective pilot study involving 44 patients. All surgeries were performed by the same surgeon. All patient received the same implant (Persona Partial Knee, ZimmerBiomet, Warsaw, USA). The patients were operated in supine position without tourniquet. The standard instrumentation according to the manufacturer’s instructions was used (ZimmerBiomet, Warsaw, USA).

Prior to the tibial resection, the resection plane was verified using the pin-less verification work-flow of the navigation device (Brainlab, Munich, Germany) or corrected in case of deviation of the expected resection plane from the target value. In group A, comprising eight patients, the tibial component was implanted in the conventional orientation of 0° with respect to the longitudinal tibial axis. In group B, consisting of 26 patients, the tibial component was implanted in 3° varus. In group C, which included 10 patients, the tibial component was implanted in 3° varus without the use of navigation software.

In cases where the tibial resection was performed using conventional instrumentation without navigation, the target 3° varus angle in the coronal plane was manually adjusted using the extramedullary alignment jig supplied by the implant manufacturer (ZimmerBiomet, Warsaw, USA). The jig was set to 3° varus relative to the mechanical axis by adjusting the valgus/varus control on the cutting guide block. Proper alignment was visually verified by referencing the tibial crest and medial malleolus, which approximates the mechanical axis in the coronal plane.

All tibial bone resections were removed in one piece without fragmentation, as this was a predefined inclusion criterion for the study to ensure the integrity of 3D surface analysis. All implants were cemented. The tibial bone resections were preserved in formalin for Cone-beam computed tomography analysis using the “SCS MedSeries^®^ H22” system.

**Fig. 3:**
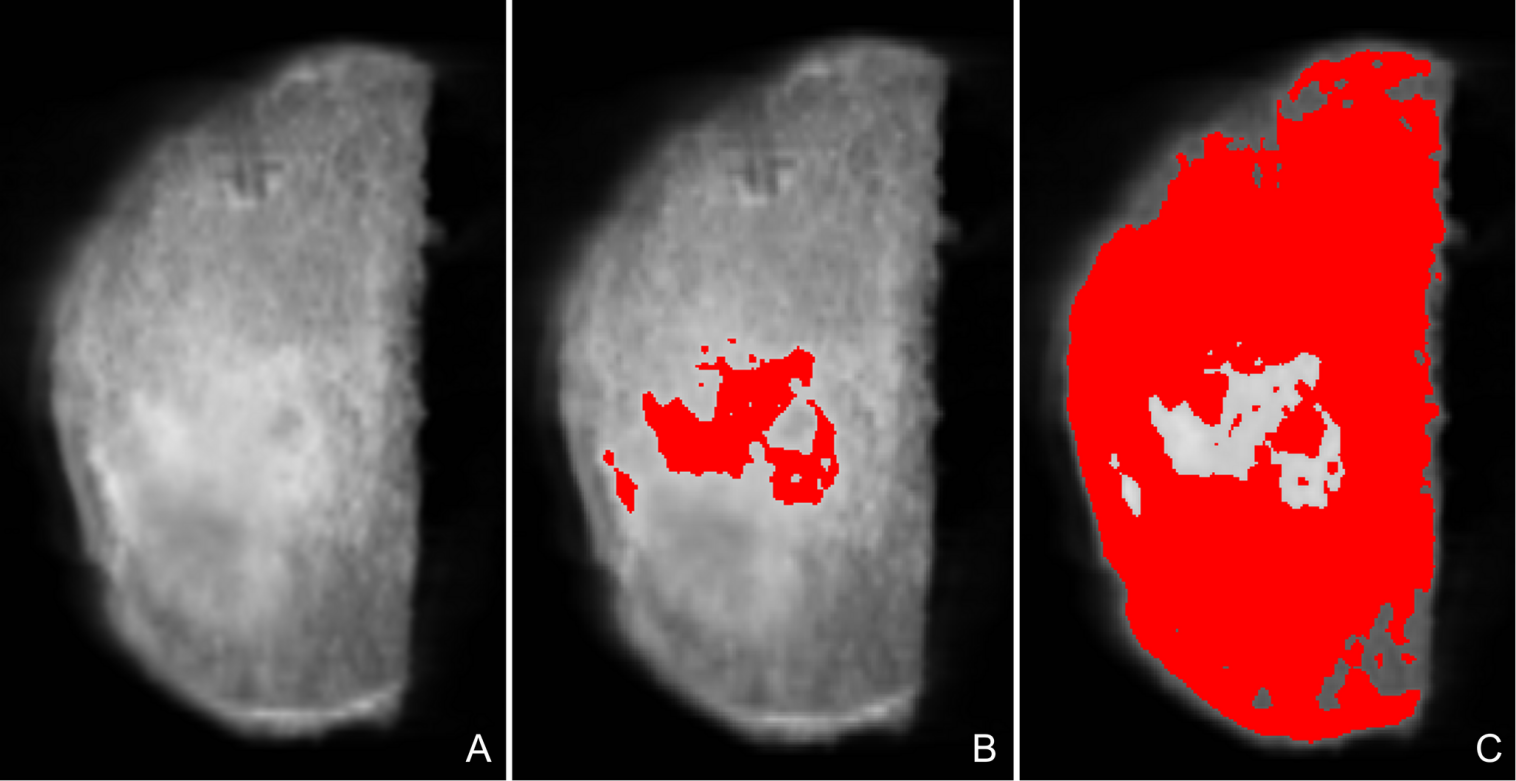
Sclerosis calculation. **A** = Native, most distal slice; **B** = Sclerosis marker; **C** = Spongious bone marker

Regarding the assessment of sclerosis percentage in the most distal slice, the slice was extracted from the CT scan after rotation correction. Subsequently, the “Interactive thresholding” and “Material statistics” tools were employed to determine the number of pixels corresponding to sclerosis and spongious bone. To establish a distinction between bone and sclerosis, a cut-off value was set at the upper third of the grey values, based on observations made by an experienced clinician (refer to Fig. 3). It should be noted that there is no definitive cut-off value available in the literature specifically for Cone-beam computed tomography images [10].

### Patient characterization

In our pilot study, we conducted an evaluation of a total of 44 tibial resections following unicompartmental knee arthroplasty. Among the participants, 8 patients underwent saw cuts in 0° varus, while 36 patients underwent cuts in 3° varus. The patient demographics are presented in Table 1. In total, the study included 24 females and 20 males. All patients in the 0° group underwent surgery on their right knee, while in the 3° group, 21 patients had surgery on their right knee and 15 patients on their left knee. Within the 0° group, 2 out of 8 patients had a history of previous arthroscopic surgery, whereas in the 3° group, 17 out of 36 patients had undergone one arthroscopic operation and 2 out of 36 patients had undergone 2 operations. The specific components (tibia, femur, and inlay) used are listed in Table 1.

**Table 1:** Patient characterization

	All N = 44	0° N = 8	3° N = 36
Sex			
Female	24	7	17
Male	20	1	19
**Side**			
Right	29	8	21
Left	15	0	15
**Previous arthroscopic operations**			
0	23	6	17
1	19	2	17
2	2	0	2
**Tibial Component**			
D	2	1	1
E	8	4	4
F	7	1	6
G	11	2	9
H	10	0	10
I	0	0	0
J	6	0	6
**Inlay-Thickness**			
8mm	19	5	14
9mm	20	3	17
10mm	5	0	5
**Femoral Component**			
3	8	6	2
4	15	1	14
5	12	1	11
6	5	0	5
7	4	0	4

Columns indicate the angle of tibial resection. The values are given as the number of cases

### Statistical analysis

The statistical analysis was conducted using SPSS Statistics Version 25 (SPSS Inc., IBM, Chicago, IL, USA). Statistical analysis was performed using the t-test to compare the means between different groups. The significance threshold was set at 0.05. Results are presented as means with standard deviations.

## Results

### Matching parameters

Table 2 presents the findings of the three-dimensional (3D) matching analysis. The distance between the surfaces of the bone resection and the implant did not exhibit a significant difference. However, the surface volumes demonstrated that the bony specimen sawed at 3° varus were larger than those with a 0° resection. Nevertheless, when comparing the ratios of the surface volumes between the bone and implant, no significant differences were observed.

**Table 2:** Matching-Parameters

	All		0°		3°		*p*-value
Surface Distance	1.8	± 0.4	1.7	± 1.4	1.8	± 1.4	0.2
Volume Bone	8161.9	± 2266.1	6641.2	± 1593.3	8499.8	± 2270.5	0.02*
Volume Implant	4241.7	± 533.3	3782.2	± 346.9	4343.8	± 516.0	0.003*
Volume Bone / Implant	1.9		1.8		1.9		0.1

Columns indicate the angle of tibial resection. The values are given as mean voxels with standard-deviations and p-value

### Location of variability

Table 3 presents the results regarding the location of variability. A significant difference between the groups was observed only for the anteromedial-central compartment, where the 3° group exhibited more discrepancies in relation to the CAD model compared to the 0° group. While the 3° group demonstrated a lower ratio of central to medial mismatches, which would suggest a more rectangular-shaped bone resection, this finding did not reach statistical significance in our small study cohort.

**Table 3:** Location of variability between bone and implant in maximal voxels

	All		0°		3°		*p*-value
Total AMM	4.1	± 1.1	3.6	± 0.9	4.3	± 1.1	0.07
Total AMC	7.1	± 1.5	5.9	± 1.7	7.3	± 1.3	0.006*
Total PMM	3.9	± 1.5	3.4	± 1.3	4.0	± 1.5	0.1
Total PMC	5.4	± 1.8	5.4	± 0.9	5.4	± 1.9	0.5
Total Mismatch	5.7	± 1.7	4.9	± 1.6	5.9	± 1.7	0.07
Quotient Central / Medial Mismatch	1.6	± 0.5	1.8	± 0.8	1.6	± 0.4	0.3

The values are given as means with standard-deviations and p-value. AMM = Anteromedial-Medial, AMC = Anteromedial-Central, PMM = Posteromedial-Medial, PMC = Posteromedial-Central

### Sclerosis

The mean percentage of sclerosis was 2.9% ± 4.9% in our study collective and did not significantly vary between the groups, with the percentage being 3.8% ± 5.7% in the 0° group and 2.7% ± 4.8% in the 3° group.

## Discussion

The objective of our study was to develop a novel method for evaluating the optimal positioning of the tibial component in unicompartmental knee arthroplasty (UKA). Furthermore, we conducted a pilot study by analyzing the bone resections after performing the tibial saw cuts in the coronal plane at 0° and 3° varus angles. We could show a good practicability of our proposed technique using the program Amira.

We had hypothesized that a 90° resection in the frontal plane would result in a wedge-shaped resection due to the preoperative medial slope of the joint line. This hypothesis could not be validated by our pilot study, either due to too small a difference between the groups, which could not be proven by our pilot group, or due to a lack of correlation. Further studies with a larger cohort group will be necessary to answer the question of the optimal tibial resection plane.

Previous computer simulations by Sekiguchi et al. and Innocenti et al. demonstrated that slight varus alignment in UKAs could improve kinematics [8], [11]. This finding was supported by Vasso et al., who conducted a clinical study using the International Knee Society knee and function scores to assess post-UKA function in a patient cohort with a mean follow-up of 7.6 years. They found that minor varus alignment yielded better IKS scores compared to neutral or close-to-neutral alignment [7]. Preserving the pre-arthritic joint line was highlighted as crucial in preventing UKA implant failure by Zambianchi et al. [12]. Collier et al. showed that a significant reduction in medial tibial joint line varus angulation resulted in higher revision rates [13].

Additionally, we examined the percentage of sclerosis in the distal cut surface to assess any differences between the groups in terms of sclerosis undercutting. Due to the limited group size of our pilot study, we could not show any significant differences between our group. However, we believe that the evaluation of the percentage of sclerosis in the distal cut surface is of clinical relevance and should be further evaluated, since intact spongious bone is essential to favor the ingrowth of the prosthesis.

This study has several limitations to consider. The sample size of 44 patients, while providing useful initial data, may limit the generalizability of the findings. Additionally, as the first study of its kind, it lacks a validation process to confirm the reliability of the methods used. Future research should include a larger cohort and incorporate a validation process to ensure the robustness of the findings. The assessment was conducted by a single blinded rater, which could introduce some subjective bias; involving multiple raters in future studies could enhance reliability. Furthermore, the tibial slope was not evaluated in our study. Given its known relevance in joint kinematics and prosthetic alignment, this represents a limitation. Future investigations should include sagittal plane parameters such as tibial slope. Finally, the cut-off value for distinguishing bone from sclerosis in Cone-beam CT images was based on clinician observation, suggesting the need for standardized values in future research to improve reproducibility.

In conclusion, we believe that the 3D modeling technique presented holds significant potential and merits application to a larger cohort of tibial bone resections for a more comprehensive investigation into the optimal tibial resection plane. Moreover, this methodology could be instrumental in the development of patient-specific implants.

Table legends.

## Data Availability

Data is available from the corresponding author upon reasonable request.
